# Cognition and brain iron deposition in whole grey matter regions and hippocampal subfields

**DOI:** 10.1111/ejn.15838

**Published:** 2022-10-20

**Authors:** Holly Spence, Chris J. McNeil, Gordon D. Waiter

**Affiliations:** ^1^ Aberdeen Biomedical Imaging Centre, Institute of Medical Sciences University of Aberdeen Aberdeen UK

**Keywords:** cognition, hippocampal subfields, iron, MRI, QSM

## Abstract

Regional brain iron accumulation is observed in many neurodegenerative diseases, including Alzheimer's disease and Parkinson's disease, and is associated with cognitive decline. We explored associations between age, cognition and iron content in grey matter regions and hippocampal subfields in 380 participants of the Aberdeen children of the 1950s cohort and their first‐generation relatives (aged 26–72 years). Participants underwent cognitive assessment at the time of MRI scanning. Quantitative susceptibility mapping of these MRI data was used to assess iron content in grey matter regions and in hippocampal subfields. Principle component analysis was performed on cognitive test scores to create a general cognition score. Spline analysis was used with the Akaike information criterion to determine if order 1, 2 or 3 natural splines were optimal for assessing non‐linear relationships between regional iron and age. Multivariate linear models were used to assess associations between regional iron and cognition. Higher iron correlated with older age in the left putamen across all ages and in the right putamen of only participants over 58. Whereas a decrease in iron with older age was observed in the right thalamus and left pallidum across all ages. Right amygdala iron levels were associated with poorer general cognition scores and poorer immediate recall scores. Iron was not associated with any measures of cognitive performance in other regions of interest. Our results suggest that, whilst iron in some regions was associated with cognitive performance, there is an overall lack of association between regional iron content and cognitive ability in cognitively healthy individuals.

## Graphical abstract

We explore associations between age, cognitive abilities and iron content in grey matter regions and hippocampal subfields in 380 participants of the Aberdeen children of the 1950s cohort and their first‐generation relatives (aged 26–72 years). Our results suggest that, whilst iron in some regions was associated with cognitive performance, there is an overall lack of association between regional iron content and cognitive ability in cognitively healthy individuals within this age group.

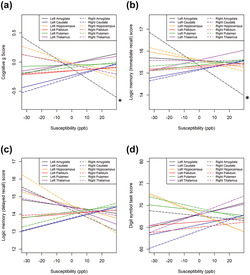

**Abbreviations**
ACONF: Aberdeen children of the 1950s
AIC: Akaike information criterion
BET: brain extraction tool
CA: Cornu Ammonis
FA: flip angle
FOV: field of view
MEGRE: multi‐echo gradient‐echo
MRI: magnetic resonance imaging
QSM: Quantitative Susceptibility Mapping
STRADL study: STratifying Resillience and Depression Longitudinally study
TE: echo time
TI: inversion time
TR: repetition time

## 1 INTRODUCTION

Iron is the most abundant transition metal in the human brain, which is essential for many biological processes, including as a necessary cofactor for enzymes involved in dopamine and serotonin synthesis, mitochondrial function, ATP and DNA synthesis and neurotransmitter cycling (Hare et al., 2013; Ward et al., 2014). Brain iron is primarily stored in ferritin proteins, which can each hold up to 4500 Fe^3+^ ions (Michaelis et al., 1943). In healthy adults, brain iron accumulates heterogeneously with age, an occurrence mainly observed in deep grey matter nuclei (Li et al., 2014; Ward et al., 2014). This accumulation can be observed in vivo using MRI techniques based on the magnetic susceptibility of tissues, with iron stored in ferritin being the main source of susceptibility in grey matter (Haacke et al., 2015).

Quantitative susceptibility mapping (QSM) is an MRI processing technique that integrates the microscopic effects of magnetisation on tissues to generate a bulk susceptibility map, allowing for the description of the spatial distribution of tissue susceptibility (Haacke et al., 2015; Reichenbach et al., 2015). Susceptibility can be affected by the presence of myelin, calcification, iron and other transition metals (Duyn & Schenck, 2017; Haacke et al., 2015). However, due to the relatively high abundance of iron in grey matter regions compared to these other contributing factors; QSM provides a quantitative measure of iron distribution throughout the grey matter of the brain (Haacke et al., 2005). This has been validated via post‐mortem studies, which show a linear correlation between susceptibility calculated using QSM and chemically determined brain iron (Hallgren & Sourander, 1958; Langkammer et al., 2012; Sun et al., 2015).

Regional iron accumulation exceeding that seen in healthy ageing is associated with impairment in region‐related function and functional connectivity (Spence et al., 2020; Ward et al., 2014; Zachariou et al., 2020). While these associations between regional brain iron and cognitive decline are most notable in individuals with neurodegenerative disease, higher brain iron in cognitively healthy individuals has also been shown to correlate with age‐related cognitive decline in some regions (Kalpouzos et al., 2017; Rodrigue et al., 2020; Spence et al., 2020). However, in cognitively healthy populations, these associations are less well defined than those with neurodegenerative disease.

Although the mechanisms of this association between brain iron and cognitive function are unknown, it is thought that brain iron accumulates either as a cause or consequence of neurodegeneration. Current theories centre around the ability of iron to perpetuate oxidative stress via Fenton's reaction. Here, ferrous ions interact with hydrogen peroxide to form ferric ions, hydroxide ions and hydroxyl radicals–toxic reactive oxygen species with high reactivity and short diffusion distance (Hare et al., 2013; Muhoberac & Vidal, 2013; Ward et al., 2014). Increased cellular iron can also lead to ferroptosis, an iron‐mediated cell death which occurs when iron‐dependent lipid peroxidation is at exceedingly high levels, causing an innate immune response leading to tissue destruction, elevated iron via cellular iron release, increased lipid peroxidation and mitochondrial dysfunction (Muhoberac & Vidal, 2013; Proneth & Conrad, 2018). In neurodegenerative diseases, iron has been found localised to protein aggregates such as amyloid beta and tau, and in vitro studies suggest that iron is capable of amplifying amyloid beta aggregation and enhancing its toxicity (Hare et al., 2013; Smith et al., 2010). Each of these potential mechanisms could lead to brain cell death, impairing regional function and thus leading to a change in cognitive ability. Whilst these mechanisms are not explored in this study, they highlight the potential impact of brain iron accumulation on cognition.

It is not yet clear whether these relationships occur in individuals without neurological or neurodegenerative disease. Therefore, this study uses QSM as a measure of brain iron to investigate the relationships between brain iron and cognitive ability in cognitively healthy adults. Previously, more iron in the caudate, putamen and pallidum have all been shown to correlate with poorer general cognition, while iron in the hippocampus and thalamus have been shown to correlate with poorer memory performance, and iron in the basal ganglia has been shown to correlate with poorer processing speed (Spence et al., 2020). Therefore, we focused on measures of general cognition, memory and processing speed in this study, including the amygdala, caudate, hippocampus, pallidum, putamen and thalamus as regions of interest. Further, it has been suggested that some hippocampal subfields may be more sensitive to pre‐dementia change in structure than global hippocampal measures, and so we also investigate the relationships between cognitive measures and iron in the hippocampal subfields of cornu Ammonis (CA)1, CA3, CA4, subiculum and molecular layer of the hippocampus (Guo et al., 2020; Skouras et al., 2020).

## 2 METHODS

### 2.1 Participants

**FIGURE 1:**
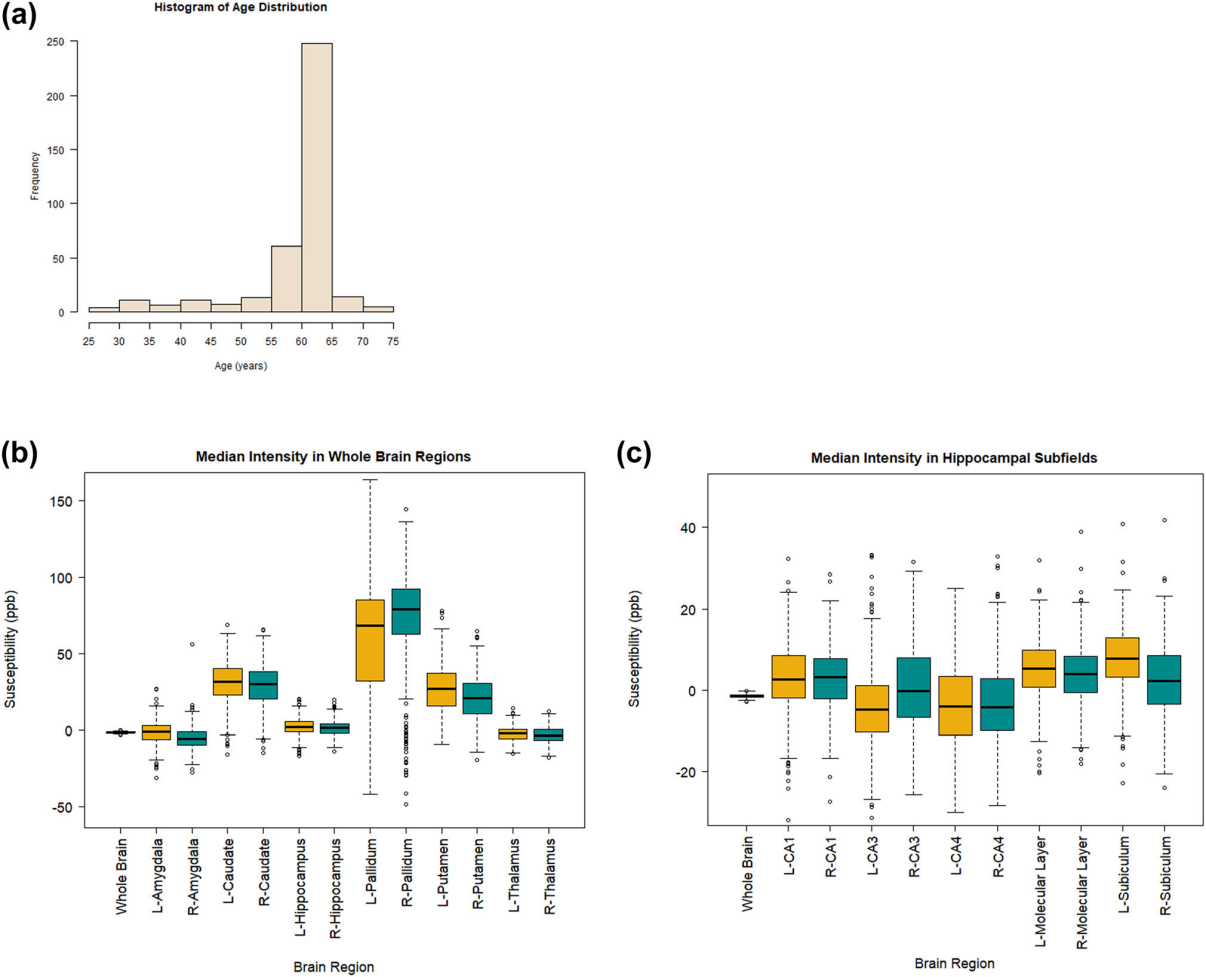
a) Histogram to show the age distribution of the 380 participants of this study. Ages ranged from 26 to 72 years old. b) Boxplot showing iron content, measured as susceptibility (ppm) using quantitative susceptibility mapping, in each whole region of interest. L = left hemisphere, R = right hemisphere. c) Boxplot showing iron content, measured as susceptibility (ppm) using quantitative susceptibility mapping, in each hippocampal subfield. L = left hemisphere, R = right hemisphere.

Participants of the Stratifying Resilience and Depression Longitudinally (STRADL) study, who were members of the Aberdeen children of the 1950s cohort or their first‐generation relatives and had complete cognitive assessment data and MRI data compatible with QSM processing were included in this study. A total of 380 participants (mean age = 59.8 ± 7.9 years; age range = 26 to 72 years) fitting these criteria were included in this study, consisting of 207 females and 173 males. Age distribution of participants is shown in Figure 1a. Participants were cognitively healthy at the time of testing. Here, ‘cognitively healthy’ refers to having no self‐reported diagnosed neurodegenerative disease at the time of assessment.

### 2.2 Imaging

MRI was performed following the protocol described by Habota et al. (2019). For our current study, only T1 weighted, T2 weighted and multi‐echo gradient‐echo images (MEGRE) were used. They were acquired on a 3 T Philips Achieva TX‐series MRI system (Philips Healthcare, Best, Netherlands) with a 32‐channel phased‐array head coil. 3D T1‐weighted fast gradient echo images were acquired with the following parameters: 160 sagittal slices, TR = 8.2 ms, TE = 3.8 ms, TI = 1031 ms, FA = 8°, FOV = 240 mm, matrix size = 240 × 240, voxel size = 1.0 × 1.0 × 1.0 mm^3^, acquisition time = 5 min 38 s. T2 weighted images were acquired with the following parameters: 360 sagittal slices, TR = 2500 ms, TE = 314 ms, FA = 90°, FOV = 250 mm, matrix size = 252 × 250, voxel size = 0.5 × 0.5 × 0.5mm^3,^ acquisition time = 7 min 17 s. MEGRE images were acquired with the following parameters: 130 axial slices, TR = 31, TE = 7.2/13.4/19.6/25.8 ms, FA = 17°, FOV = 230 mm, matrix size = 384 × 316, Voxel size = 0.3 × 0.3 × 1 mm^2^, acquisition time = 4 min 29 s.

**FIGURE 2:**
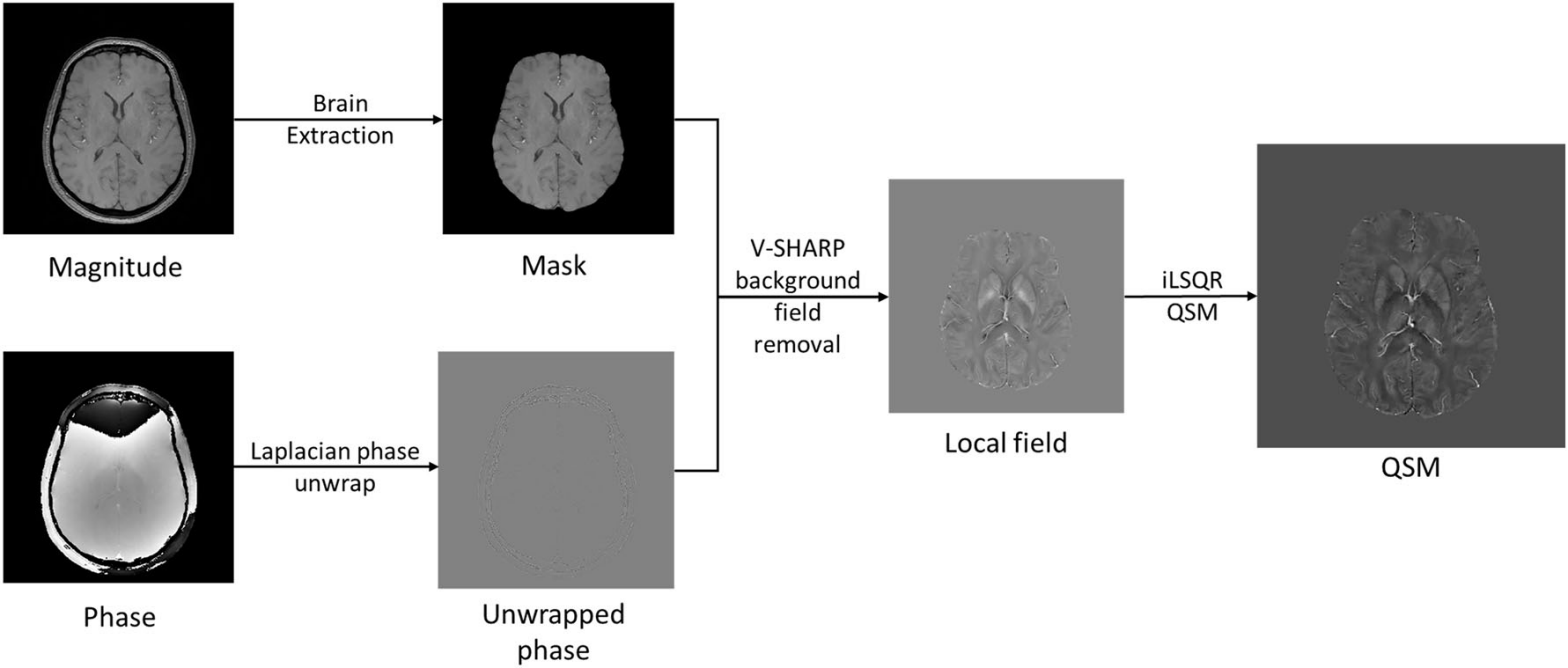
Schematic to show quantitative susceptibility mapping processing pipeline used to convert multi‐echo gradient‐echo (MEGRE) images into a susceptibility map using STI suite v3.0.

Phase images from MEGRE data were processed through a quantitative susceptibility mapping (QSM) pipeline to calculate susceptibility maps used to measure iron content. Brain masks were generated using the brain extraction tool (BET) function of FSL (Smith, 2002). The STISuite V3.0 (Li et al., 2013) QSM GUI pipeline was used with Laplacian based phase unwrapping, a VSHARP background field correction and the iLSQR QSM method. Figure 2 shows the complete QSM pipeline. Susceptibility maps were co‐registered with previously segmented T1‐weighted images, from which mean volume was calculated for the regions of interest, amygdala, caudate, hippocampus, pallidum, putamen and thalamus. Regions of interest were chosen based on regions that were previously shown to have associations with cognition (Spence et al., 2020). Segmentation and regional volume analyses for these regions of interest were carried out using publicly available FreeSurfer software with the Desikan‐Killiany atlas (Fischl, 2012). Hippocampal subfields cornu Ammonis (CA)1, CA3, CA4, subiculum, and molecular layer of the hippocampus were also included as regions of interest. For hippocampal subfields, T2 weighted images were segmented using the method previously described by Iglesias et al. (2015) and these segmented images were co‐registered with susceptibility maps to calculate mean volume (Iglesias et al., 2015). All regions of interest had left and right regional volume and susceptibility calculated separately. As regional susceptibility data was skewed, regional median susceptibility was calculated for each region of interest as a measure of regional iron content.

### 2.3 Cognitive assessment

A series of cognitive tests were used to assess cognitive ability and are described in the STRADL cohort profile (Habota et al., 2019). Briefly, the Mill Hill vocabulary test was used to measure verbal intelligence and the controlled word association task was used to measure verbal fluency (Benton, 1994; Raven, 1989). A UK version of the logical memory subtest of the Wechsler adult intelligence scale III was used to measure immediate and delayed verbal memory and recall (Wechsler, 1997). To measure processing speed, the digit symbol coding task of the Wechsler adult intelligence scale III was performed (Wechsler, 1997). Finally, a paper version of the COGNITO psychometric examination, the matrix reasoning test, was performed to examine perceptual organisation and visuospatial logic (Ritchie et al., 2014).

### 2.4 Statistical analyses

**FIGURE 3:**
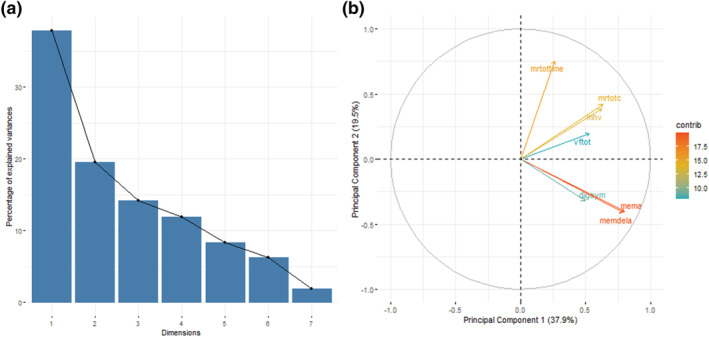
a) Scree plot to show the variance explained by each principal component. The first principal component (dimension 1) was used in this study as a measure of general cognition. b) Biplot shows the loadings of each cognitive variable on the first principal component.

All statistical analyses were carried out using R software (R core team, 2021). Principal component analysis was applied to cognitive assessment scores (Mill Hill vocabulary score, controlled word association task total score, logical memory immediate score and delayed score, digit symbol coding task score, matrix reasoning total correct score and time taken) to generate a g score for general cognition (g). The g score was defined as the first principal component in this analysis. Figure 3 shows the loadings of the principal component used. Due to previously reported non‐linearity of iron deposition with age, we used natural splines (ns in ‘splines’ r package) to determine the relationships between susceptibility and age. The Akaike information criterion (AIC) was used to determine whether order 1, 2 or 3 splines best fit the data and, subsequently, whether sex should be included as a confounding factor. The spline with the lowest AIC was reported as the best fit. To examine associations between susceptibility and cognitive scores, linear regression modelling was used. Due to the previously reported differences in cognition depending on age, regional volume, education and differences in iron status between sexes, we corrected for these covariates in regression analyses. An AIC was used to determine whether confounding factors significantly contributed to the model. Variables were included if a decrease in AIC score was observed when the variable was added to the model, indicating less information was lost by the model. No multiple comparison correction was used in the analysis of whole grey matter regions, as the hypotheses tested for these regions were decided a priori following a systematic review (Spence et al., 2020). For the analysis of hippocampal subfields, a Holm‐Bonferroni multiple comparison correction was applied, with a corrected α = 0.05 denoting significance.

## 3 RESULTS

### 3.1 Regional Iron concentrations

Figure 1 shows the susceptibility values for each of the whole regions and hippocampal subfields assessed with whole brain susceptibility included for reference. Average susceptibility is reported here as a mean ± standard deviation. Average whole brain susceptibility was −1.36 ± 0.481 ppb. Of the whole regions of interest assessed, the pallidum had the highest iron content in both left and right hemispheres (56.9 ± 40.3 ppb and 72.5 ± 32.7 ppb respectively), followed by the caudate (31.0 ± 14.4 ppb and 29.2 ± 14.1 ppb respectively), putamen (26.7 ± 16.7 ppb and 20.5 ± 14.7 ppb respectively) and then the hippocampus (2.32 ± 5.82 ppb and 1.35 ± 5.66 ppb respectively). The regions with the lowest iron content were the thalamus (−2.20 ± 4.91 ppb and −3.04 ± 5.43 ppb respectively) followed by the amygdala (−1.44 ± 7.94 ppb and −5.08 ± 7.57 ppb respectively).

Of the hippocampal subfields in the left hemisphere, the subiculum had the highest iron content (7.59 ± 8.23 ppb) followed by the molecular layer (5.14 ± 7.22 ppb), CA1 (3.00 ± 8.93 ppb), CA4 (−3.83 ± 10.2 ppb) and CA3 (−4.16 ± 10.2 ppb). In the right hemisphere, the molecular layer had the highest iron content (4.28 ± 7.22 ppb) followed by the CA1 (2.91 ± 7.31 ppb), subiculum (2.55 ± 8.93 ppb), CA3 (1.03 ± 10.5 ppb) and CA4 (−3.49 ± 10.46 ppb).

### 3.2 Regional Iron and age

**FIGURE 4:**
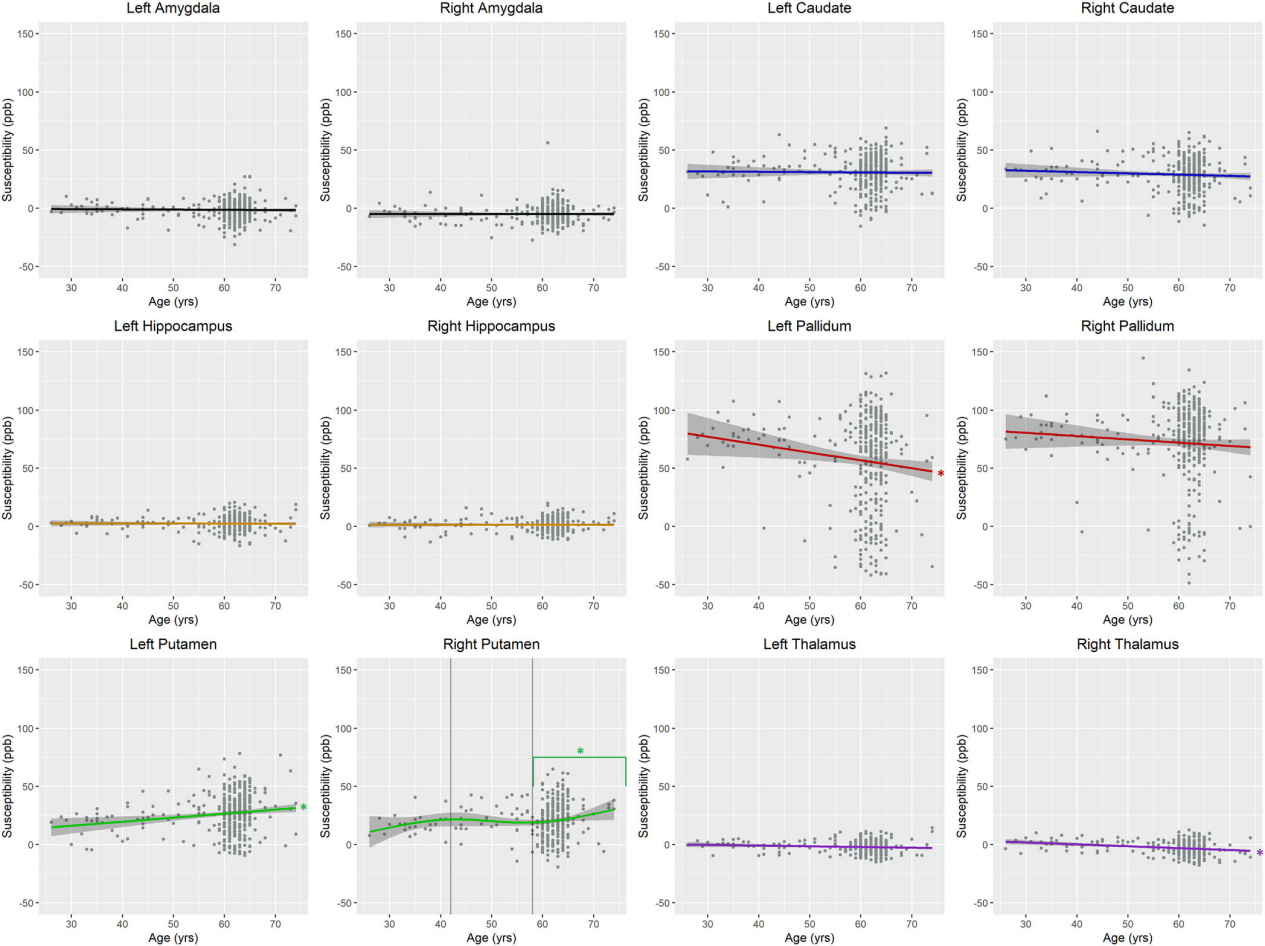
Relationships between iron content in grey matter regions as determined by quantitative susceptibility mapping on MRI and age at time of scan. * Indicates associations that were statistically significant.

**FIGURE 5:**
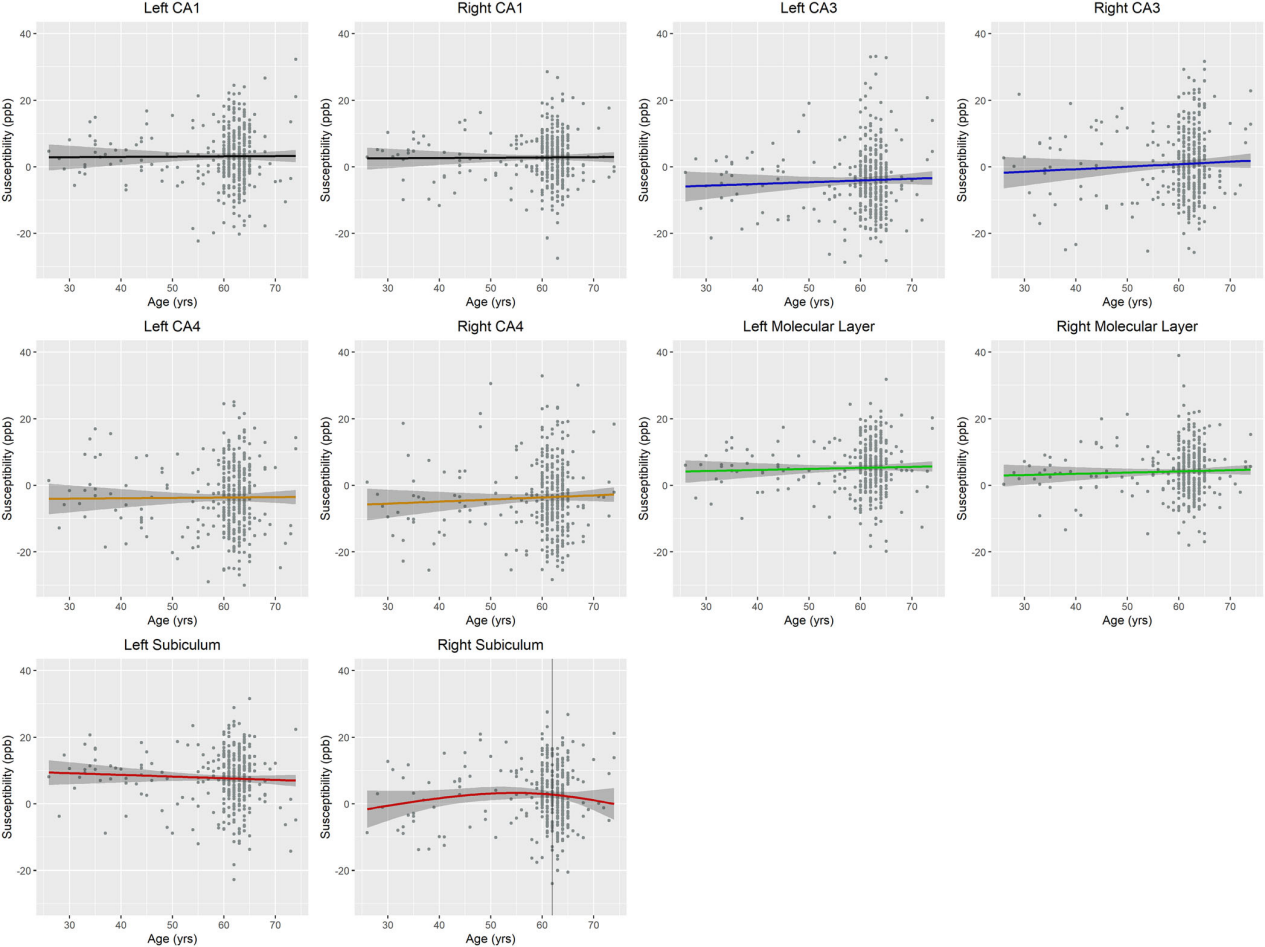
Relationships between iron content in hippocampal subfields as determined by quantitative susceptibility mapping on MRI and age at time of scan. None of these interactions reached statistical significance.

The ‘best fit’ models comparing age and susceptibility are presented in Table 1. Iron in the left putamen significantly positively correlated with age across all ages (p = 0.001, β = 0.162, R^2^ = 0.026). This association remained significant after controlling for sex (p = 0.001, β = 0.164, R^2^ = 0.036). Higher iron in the right putamen was also associated with older age only in those over 58 years old (p = 0.026, β = 0.130, R^2^ = 0.007). This association remained significant after controlling for sex (p = 0.016, β = 0.141, R^2^ = 0.014). However, iron in the left pallidum and right thalamus negatively correlated with age (p = 0.010, β = −0.132, R^2^ = 0.015; p < 0.001, β = −0.236, R^2^ = 0.053 respectively). These associations remained significant after correcting for covariates (p = 0.005, β = −0.149, R^2^ = 0.024; p < 0.001, β = −0.236, R^2^ = 0.053 respectively). Iron was not significantly associated with age in any other regions of interest (Table 1; Figures 4 and 5).

**TABLE 1:** Summary of linear regression analysis between regional iron content (as measured by quantitative susceptibility mapping) and age

Region of interest	Order spline	Covariate	Order 1 β(26–74 years)	Order 1 P(26–74 years)	Order 2 β(26–62 years)	Order 2 p(26–62 years)	Order 2 β(62–74 years)	Order 2 p(62–74 years)	Order 3 β(26–42 years)	Order 3 P(26–42 years)	Order 3 β(42–58 years)	Order 3 p(42–58 years)	Order 3 β(58–74 years)	Order 3 p(58–74 years)
Left amygdala	1		−0.019	0.716										
Right amygdala	1		0.003	0.952										
Left caudate	1	Sex/Vol	−0.035	0.514										
Right caudate	1	Sex	−0.059	0.242										
Left Hippocampus	1	Sex/Vol	−0.023	0.657										
Right Hippocampus	1	Sex	−0.006	0.908										
Left pallidum	1	Sex/Vol	↓ −0.149	0.005*										
Right pallidum	1	Sex	−0.066	0.197										
Left putamen	1	Sex	↑ 0.164	0.001*										
Right putamen	3	Sex							−0.021	0.716	0.108	0.086	↑ 0.141	0.016*
Left thalamus	1	Sex	−0.099	0.052										
Right thalamus	1		↓ −0.236	<0.001*										
Left CA1	1	Sex	0.007	0.897										
Right CA1	1	Sex/Vol	0.024	0.642										
Left CA3	1	Sex	0.039	0.437										
Right CA3	1	Sex	0.054	0.281										
Left CA4	1	Sex/Vol	0.024	0.642										
Right CA4	1	Sex	0.044	0.380										
Left molecular layer	1	Sex	0.032	0.533										
Right molecular layer	1	Sex/Vol	0.032	0.523										
Left subiculum	1		−0.049	0.345										
Right subiculum	2	Sex			0.088	0.092	−0.056	0.287						

↓ = negative correlation, ↑ = positive correlation.

### 3.3 Regional Iron and g scores

**FIGURE 6:**
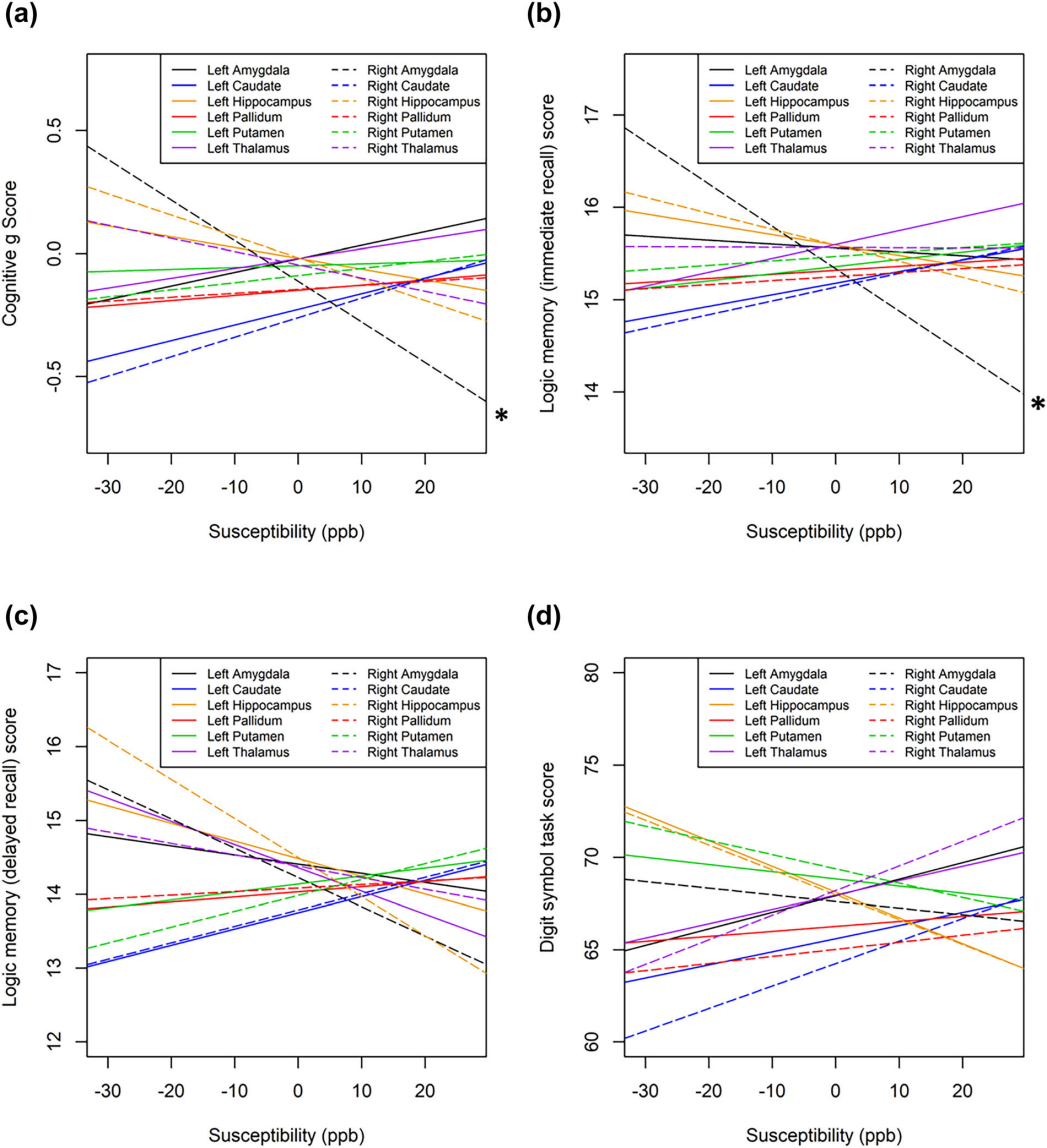
a) Relationships between general cognition and iron content in grey matter regions as determined by quantitative susceptibility mapping on MRI. b) Relationships between performance in the immediate verbal recall task of logical memory subtest of the Wechsler adult intelligence scale III and iron content in grey matter regions as determined by quantitative susceptibility mapping on MRI. c) Relationships between performance in the delayed verbal recall task of logical memory subtest of the Wechsler adult intelligence scale III and iron content in grey matter regions as determined by quantitative susceptibility mapping on MRI. d) Relationships between performance in the digit symbol coding task of the Wechsler adult intelligence scale III and iron content in grey matter regions as determined by quantitative susceptibility mapping on MRI. * indicates interactions that were statistically significant.

**FIGURE 7:**
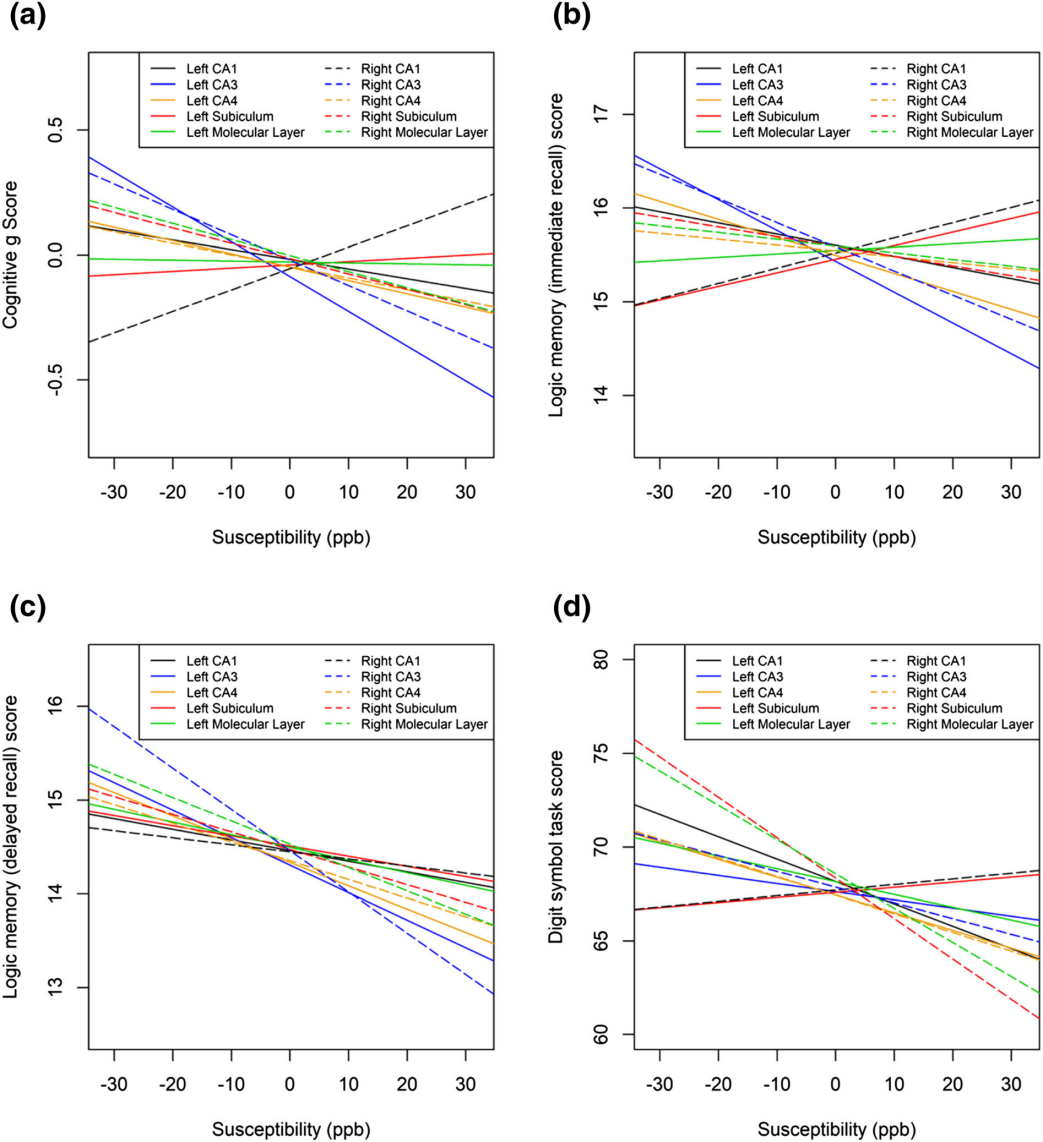
a) Relationships between general cognition and iron content in hippocampal subfields as determined by quantitative susceptibility mapping on MRI. b) Relationships between performance in the immediate verbal recall task of logical memory subtest of the Wechsler adult intelligence scale III and iron content in hippocampal subfields as determined by quantitative susceptibility mapping on MRI. c) Relationships between performance in the delayed verbal recall task of logical memory subtest of the Wechsler adult intelligence scale III and iron content in hippocampal subfields as determined by quantitative susceptibility mapping on MRI. d) Relationships between performances in the digit symbol coding task of the Wechsler adult intelligence scale III and iron content in hippocampal subfields as determined by quantitative susceptibility mapping on MRI.

Iron in the right amygdala significantly negatively correlated with g score (p = 0.047 β = −0.0884, R^2^ = 0.257) after controlling for age, sex and volume. Before controlling for covariates, this association was not significant. Iron content was not significantly associated with g score in any other whole grey matter regions of interest (Table 2; Figure 6). Interestingly, iron in hippocampal subfields was not associated with g score in any regions. However after controlling for covariates, iron in the right CA1 region positively correlated with g score (p = 0.025, β = 0.102, R^2^ = 0.262; Table 2; Figures 6 and 7 *)*. This did not remain significant after multiple comparison correction.

**TABLE 2:** Summary of linear regression analysis between regional iron content (as measured by quantitative susceptibility mapping) and cognitive assessment scores, when controlling for covariates determined to significantly contribute to the models based on the Akaike information criterion analysis

Region of interest	General cognition		Memory (immediate recall)		Memory (delayed recall)		Processing speed	
	β coefficient	*P* value	β coefficient	*P* value	β coefficient	*P* value	β coefficient	*P* value
Left amygdala	‐		‐		‐		‐	
Right amygdala	↓ −0.088	0.047	↓ −0.105	0.0273	‐		‐	
Left caudate	‐		‐		‐		‐	
Right caudate	‐		‐		‐		‐	
Left Hippocampus	‐		‐		‐		‐	
Right Hippocampus	‐		‐		‐		‐	
Left pallidum	‐		‐		‐		‐	
Right pallidum	‐		‐		‐		‐	
Left putamen	‐		‐		‐		‐	
Right putamen	‐		‐		‐		‐	
Left thalamus	‐		‐		‐		‐	
Right thalamus	‐		‐		‐		‐	
Left CA1	‐		‐		‐		‐	
Right CA1	↑ 0.102	0.025	‐		‐		‐	
Left CA3	‐		‐		‐		‐	
Right CA3	‐		‐		‐		‐	
Left CA4	‐		‐		‐		‐	
Right CA4	‐		‐		‐		‐	
Left molecular layer	‐		‐		‐		‐	
Right molecular layer	‐		‐		‐		‐	
Left subiculum	‐		‐		‐		‐	
Right subiculum	‐		‐		‐		↓ −0.095	0.047

↓ = negative correlation, ↑ = positive correlation.

### 3.4 Regional Iron and individual cognitive test scores

After controlling for age, sex, education and volume, iron in the right amygdala was significantly negatively correlated with immediate recall memory scores (p = 0.027, β = −0.105, R^2^ = 0.142). Before controlling for covariates, this association only trended towards significance (p = 0.055, β = −0.098, R^2^ = 0.007). No other cognitive task scores were correlated with iron content in any other whole region of interest (Table 2; Figure 6). Right subiculum iron content correlated negatively with digit symbol task score for processing speed (p = 0.007, β = −0.138, R^2^ = 0.017), remaining significant after correcting for covariates (p0.047, β = −0.142, R^2^ = 0.0175). However, this did not remain significant after the multiple comparison correction. Iron in other hippocampal subfields was not associated with any other individual cognitive task scores (Table 2; Figure 7). No other cognitive task scores were associated with brain iron in any other regions of interest.

## 4 DISCUSSION

This study showed positive correlations between age and iron content in the left putamen and in the right putamen of only older adults (58–74 years). However, negative correlations between iron content and age were observed in the left pallidum and right thalamus. When looking at iron levels across different grey matter regions, we showed that iron in the pallidum had much higher variability compared to all other grey matter regions. This suggests that the pallidum may be more susceptible to the involvement of environmental factors in iron metabolism. When comparing iron content and cognitive ability, higher iron in the right amygdala was associated with poorer general cognition and immediate recall memory scores. Iron in other regions of interest, including hippocampal subfields, was not associated with any measures of cognition.

Previous studies show that iron in the brain increases up to around 20 years old, where it plateaus until rapid accumulation resumes at around 60 years old (Hallgren & Sourander, 1958; Larsen et al., 2020; Li et al., 2014). We therefore assessed differences in regional iron with age using order 1–3 natural splines as changes in brain structures with age likely follow a non‐linear relationship with a more rapid rate of change occurring after 60 years old (Bussy et al., 2021). While linear models best fit our data in most regions, we showed that a 3rd order natural spline best fitted the relationships between iron in the right putamen and age. Here, an increase in iron with age was observed in the older age group (58–74 years old), which suggests that iron may only start to rapidly accumulate in later life in some regions.

Our finding that only iron in the putamen increases with age is consistent with other similar studies, while some studies looking at iron deposition in cognitively healthy participants also found increases in iron with age in other regions, particularly in the caudate and hippocampus (Howard et al., 2020; Rodrigue et al., 2020; van Bergen et al., 2018; Venkatesh et al., 2021; Zachariou et al., 2021). The observed decrease in iron in the pallidum and thalamus with age is not consistent with other studies where iron in these regions either increases with age or shows no association with age (Spence et al., 2020; Venkatesh et al., 2021). The mean age of participants in this study was 60 years old, and it could be that participants around this age have not reached the stage of ageing where iron begins to rapidly increase. Furthermore, while the age range of this study was from 26 to 72 years old, the majority of participants were aged between 55 and 65 years old (Figure 1a). This is due to the nature of the data collected from the Aberdeen children of the 1950s birth cohort. This limited spread of data could further impede our ability to distinguish differences in iron across the lifespan.

We also observe weak but significant associations between higher iron in the right amygdala and poorer general cognition scores and poorer performance in the immediate recall memory task. Given that the amygdala is involved in memory processing, such relationships between iron in this region and memory performance would agree with our previous work that suggests that higher regional iron can affect cognitive abilities relating to the function of said region (Spence et al., 2020). Although the iron content was significantly higher in older adults, putamen iron levels did not correlate significantly with any measures of cognition. Other studies looking at cognitively healthy participants have shown inverse correlations between regional iron content and performance in cognitive tasks that were not observed in this study, particularly in the hippocampus and putamen (Chen et al., 2021; Venkatesh et al., 2021; Zachariou et al., 2021). However, there is disagreement among some studies in cognitively healthy individuals as to the regions in which such relationships are observed. For example, while Ghadery et al. (2015) demonstrated an association between higher pallidum iron and poorer cognitive performance, Darki et al. (2016) showed only a correlation between higher caudate iron and cognition and no correlation between iron in the pallidum and cognition. Put together with previous studies, our results highlight the yet poorly characterised associations between brain iron and cognition in healthy adults.

In this study, we also found that total hippocampal iron content did not correlate with performance in any cognitive task. Some very weak associations were observed between higher iron in the right subiculum and poorer processing speed, and higher iron in the CA1 subfield was weakly associated with a higher g score. However, these associations did not remain significant after multiple comparison correction and, moreover, the association between right subiculum iron and processing speed had adjusted r^2^ < 0.02, meaning less than 2% of variance was explained by this model. This is consistent with several other studies which also found no associations between total hippocampal iron and cognition (Blasco et al., 2017; Li et al., 2015). However, others have shown correlations between higher iron in the hippocampus and memory performance (Chen et al., 2021; Zachariou et al., 2021). This variation in results is likely due to the differences in design, with some studies looking across a broader age span than others. In this way, studies showing no correlation between hippocampal iron and cognition tend to include participants of younger ages (<45), while those studies that look at only older adults (>55) have shown a significant association between hippocampal iron and cognitive impairment (Blasco et al., 2017; Chen et al., 2021; Li et al., 2015; Zachariou et al., 2021). This suggests that hippocampal iron may only impact cognition in older adults.

Overall, the lack of significant associations observed in this study suggests that without distinct pathology, brain iron may not have a significant impact on cognitive ability in adults under 72 years old. There are several factors which may contribute to this general lack of interaction. Studies investigating associations between regional brain iron and cognition in neurodegenerative disease often observe that it is an increase in iron *compared to* healthy ageing that is associated with pathology or poorer cognition in disease (Ayton et al., 2019; Ding et al., 2009). It is possible that iron may accumulate more rapidly as a consequence of disease state. Therefore, in cognitively healthy participants, such as those included in this study, iron accumulation may not have reached a high enough level to impact cognitive decline. Rapid iron accumulation occurring as a consequence of neurodegeneration would explain our results showing that, in the absence of pathology, high levels of iron accumulation are not observed. It could also be that, in the absence of pathology, our participants were too young for iron‐cognition relationships to be observed. The participants of this study are all under 72, while some studies have shown that iron levels were only associated with iron in older adults in the absence of neurodegenerative disease (Chen et al., 2021; van Bergen et al., 2018).

## 5 LIMITATIONS

A potential contributor to variance in the models presented in this study that was not accounted for is the potential presence of Alzheimer's pathology in otherwise cognitively healthy participants. It is known that amyloid β42 and tau burden occur in a relatively large portion of non‐demented populations and are a risk factor for cognitive decline, mild cognitive impairment and Alzheimer's disease (Ebenau et al., 2020; Ingala et al., 2021). Several studies have shown different associations between iron and cognition in those who are amyloid positive compared to amyloid negative individuals, as well as showing direct interaction between amyloid, iron and regional volume (Ayton et al., 2017; Foster et al., 2020). Therefore, as it is likely that some participants in this study have this underlying pathology, this would be a contributor to individual differences in cognition that are not included in the models we present.

A limitation of the current study is that iron and cognition were only measured in participants at one time point. ‘Normal’ baseline iron and cognitive ability may vary between individuals, and rate of iron increase may be more indicative of abnormalities in brain metabolism. In the future, it is important for studies to assess the same participants over multiple time points, as it is possible that the rate of increase in iron within an individual over time is a more appropriate measure of iron accumulation than baseline iron. This would also allow us to measure decline in cognition within an individual, rather than baseline cognitive scores.

Another potential limitation here is that partial volume effects may occur, particularly at hippocampal‐amygdala boundaries. This may result in iron within some voxels which are part of the hippocampus being included as part of the amygdala, and vice versa. However, this would only occur at boundaries, meaning at relatively high resolution, this would be a small proportion of whole regions, and so the effect should not significantly affect results. For this reason, we used higher resolution T2W images to segment the hippocampal subfields in this study. Our use of these higher resolution images should mitigate the impact of partial volume effects on hippocampal subfield volume and iron measures. Furthermore, other factors such as myelin can interfere with susceptibility measures. However, in this study, we only looked at myelin‐poor grey matter regions where validation studies have shown that iron correlates directly with susceptibility (Hallgren & Sourander, 1958; Langkammer et al., 2012; Sun et al., 2015). This means the effects of these other materials on susceptibility in our regions of interest should be insignificant.

## 6 CONCLUSIONS

To conclude, we observed some weak relationships between iron and cognition in the right amygdala. However in other grey matter regions, our results suggest a lack of association between iron content and cognitive ability in individuals without diagnosed neurological or neurodegenerative disease. At a mean age of 60, the participants of this study may be too young to have iron accumulation at levels high enough to impact cognition in the absence of pathology, however, it is possible that the rate of iron increase within individuals is a more appropriate marker for cognitive decline in cognitively healthy individuals.

## CONFLICT OF INTEREST

The authors report no conflicts of interest.

## AUTHOR CONTRIBUTIONS

HS, CM and GW contributed to the conception and design of the study. GW and HS performed the MRI data analysis. HS performed subsequent statistical analysis and wrote the first draft of the manuscript. All authors contributed to manuscript revision, read and approved the submitted version.

### PEER REVIEW

The peer review history for this article is available at https://publons.com/publon/10.1111/ejn.15838.

## Data Availability

The data collected in the STRADL study have been incorporated in the larger Generation Scotland dataset. Non‐identifiable information from the Generation Scotland cohort is available to researchers in the United Kingdom and to international collaborators through application to the Generation Scotland Access Committee (access@generationscotland.org). Generation Scotland operates a managed data access process including an online application form, and proposals are reviewed by the Generation Scotland Access Committee.
